# Diet Quality and Adequacy of Nutrients in Preschool Children: Should Rice Fortified with Micronutrients Be Included in School Meals?

**DOI:** 10.3390/nu8050296

**Published:** 2016-05-14

**Authors:** Ceres M. Della Lucia, Kellen Cristina C. Rodrigues, Vivian Cristina C. Rodrigues, Laura Luiza M. Santos, Leandro M. Cardoso, Hércia S. D. Martino, Sylvia C. C. Franceschini, Helena Maria Pinheiro-Sant’Ana

**Affiliations:** 1 Department of Nutrition and Health, Federal University of Viçosa, Av. Purdue, Campus, Viçosa, MG 36570-900, Brazil; kellen.rodrigues.nut@gmail.com (K.C.C.R.); vivian_rodrigues2007@hotmail.com (V.C.C.R.); laura.santos@ufv.br (L.L.M.S.); hercia@ufv.br (H.S.D.M.); sylvia@ufv.br (S.C.C.F.); helena.santana@ufv.br (H.M.P.-S.); 2 Department of Nutrition, Federal University of Juiz de Fora, Av. Doutor Raimundo Monteiro Rezende, 330, Downtown, Governador Valadares, MG 35.010-177, Brazil; lcardoso.nutricao@hotmail.com

**Keywords:** vitamins, minerals, fortification, hidden hunger, health eating index

## Abstract

Feeding is indicative of the nutritional status of children, however micronutrient deficiency is common in this age group. We evaluated the impact of inclusion of rice (Ultra Rice^®^ (UR^®^)) fortified with iron, zinc, thiamin and folic acid on laboratory measurements and the nutrient intake of children. Ninety-nine preschoolers (2–6 years; 42.6% male) from two preschools participated, one of which received UR^®^ added to polished rice as part of school meals (test preschool) and the other received only polished rice (control preschool). Biochemical evaluations were performed before and after four months of intervention. Feeding was assessed by direct weighing of food, complemented by 24-h recalls, and the diet was assessed by the Healthy Eating Index (HEI) adapted to the Brazilian reality. The fortified rice improved the levels of zinc (*p* < 0.001), thiamine (*p* < 0.001), folic acid (*p* = 0.003), mean corpuscular hemoglobin (*p* < 0.001) and mean corpuscular hemoglobin concentration (*p* < 0.001). The inadequacy percentages of thiamine, folic acid and iron were lower among preschoolers from the test preschool. This study demonstrated the effectiveness of using UR^®^ on laboratory measurements of children. The inadequate intake of thiamine, folic acid and iron was also reduced, making the fortified rice an interesting strategy in school feeding programs.

## 1. Introduction

Childhood is a period of extreme importance for growth and development, and because it is a phase of intense growth, nutritional needs are increased and adequate nutrition is essential [1]. In this stage, the foods preferentially consumed are those high in fat, sugar and salt, *i.e.*, foods with high energy content and low nutritional value, while the intake of fruits and vegetables is low [2].

Such eating habits can cause disorders such as overweight and obesity and predisposition to chronic diseases including hypertension, diabetes, heart disease, osteoporosis and cancer [3]. Additionally, these habits can lead to subclinical micronutrient deficiency, also known as hidden hunger, which affects about one-third of the world population and can have both mental and physical consequences [4]. Most common are the deficiencies of iron, iodine, zinc and vitamin A [5].

The food intake assessment is a tool used to correlate a population’s eating habits with the presence of morbidity and mortality, allowing early detection of nutritional deficiencies in vulnerable groups, such as children [6].

The complexity of human feeding and its correlation to health require the creation of tools to assess diet quality. The Healthy Eating Index (HEI) is an educational and preventive instrument created by the United States Department of Agriculture, which aims to assess the changes in population dietary patterns based on nutritional recommendations. The Brazilian HEI has been adapted to the reality of the country and it evaluates the intake of nutrients and food through twelve components that are individually scored, and this score can vary between 0 and 100. High scores in HEI correlate positively to a varied diet, adequate intake of fruits, micronutrients and fiber and low consumption of total and saturated fat [7].

Diversified diet and nutrition educational tools are used together in the construction of knowledge regarding correct and balanced food choices [4,8]. As a joint strategy for the prevention of certain nutritional diseases, technology may also play an important role, since it permits the development of new ingredients and foods geared toward the health and welfare of the population [9].

Food fortification has been used and considered socially acceptable to effectively reduce micronutrient deficiencies, requiring no change in eating habits, and does not change the characteristics of the food [10]. To be suitable for fortification, a food must be regularly consumed, which makes rice an excellent choice since this is a base cereal in countries with high prevalence of specific deficiencies [10,11,12].

The fortification technology Ultra Rice^®^ (UR^®^) consists of transforming broken rice grains into rice flour which is combined with a binder with other fortifying nutrients and remodeled in rice grains of the same size, shape and texture when compared to the polished rice. The levels of fortification agents are concentrated in these grains, so it must be mixed with the polished rice at a ratio of between 1:50 and 1:200 [13].

The implementation of UR^®^ in food politics seeks to disseminate the food, especially in programs that address risk populations for such deficiencies, in this case, preschool children.

By identifying the factors associated with quality of the preschool diet, one can identify those which contribute to its adequacy and evaluate the measures to be taken to correct the inadequacies. In this sense, the objective of this study was to evaluate the need for inclusion of UR^®^ in the meals of preschool children, considering their food intake, the quality of their diet and the impact of its use on the laboratory measurements of these children.

## 2. Experimental Section

### 2.1. Raw Material

Extruded rice grains (Ultra Rice^®^) containing iron (micronized ferric pyrophosphate), zinc (zinc oxide), thiamin (thiamine mononitrate) and folic acid, produced by a pasta manufacturer (Adorella Foods Ltd.) located in Indaiatuba, Sao Paulo, Brazil, and kindly granted by Program for Appropriate Technology in Health (PATH), were used.

For rice preparation in the test preschool, the UR^®^ grains were mixed with polished rice, at a ratio of 1:100, and served at lunch. One gram of cooked UR^®^ provided two-thirds (66.66%) of the daily recommendations of iron, zinc, thiamine and folic acid for preschool age children. Thus, for example, a 50 g portion of this preparation contains 0.5 g of UR^®^ and meets a third (33.33%) of the Recommended Dietary Allowances (RDA) [14] of these micronutrients for this population group. The control preschool received conventional polished rice as part of their regular diet.

### 2.2. Sample

The sample size was calculated according to Mera *et al*. (1998) [15], considering ferritin as the main variable. A statistical power of 80%, a significance level of 5% and an expected difference of 17% in the baseline values were adopted, totaling a sample of 45 individuals in each experimental group.

**Figure 1:**
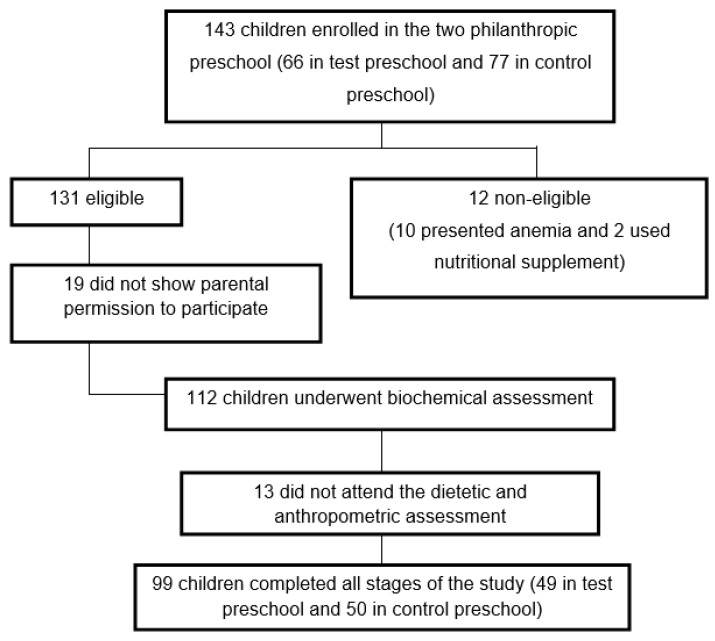
Diagram of the sample selection of children enrolled in the two philanthropic preschools.

The study was conducted in two philanthropic preschools in Brazil. One hundred and forty-three children between 2 and 6 years old, of both genders, were considered eligible to participate in the study. The eligibility criteria were: children whose hemoglobin (Hb) levels were equal to or greater than 11.0 g/dL, presenting no infectious process or inflammation, current consumers of rice and whose caretakers signed the consent form, authorizing the child’s participation in the study. Children diagnosed with iron deficiency anemia, who received ferrous sulfate supplementation or other nutritional supplements after evaluation by a qualified doctor of the city Health Department, were excluded from the study for ethical reasons since thefortifiedriceis afoodtesting. A total of 131 children were selected, of which blood was taken from 112 after obtaining parental consent. Of those, 13 did not return for the dietary and anthropometric assessments, yielding a total of 99 children who participated in all sessions of the study (Figure 1). The preschools were randomly selected as “control” or “test”.

### 2.3. Experimental Design

A four-month intervention study was conducted, in which children of the selected preschools received, as part of school meals, polished rice (control group) or UR^®^ mixed with polished rice (test group). Rice portions (50 g) were served daily, Monday through Friday, as part of the preschool lunch. Children were submitted to laboratory tests before and after the intervention period. Laboratory tests were conducted at the Laboratory of Clinical Analyses, Division of Health of the Federal University of Viçosa (UFV). Before initiating the study, children were dewormed with Albendazole (oral suspension, 40 mg), as medically indicated.

### 2.4. Laboratory Tests

Laboratory tests were conducted at the beginning and end of the intervention period. Samples containing approximately 12 mL of blood were collected in syringes by venipuncture from a brachial vein from each study participant, at the same time of the day (between 8:00 and 9:00 a.m.), to avoid diurnal variation in the evaluated parameters. About 4 mL of the sample were transferred to a tube containing anticoagulant ethylene diaminetetraacetic (EDTA) to perform full blood count and erythrocyte thiamine; 4 mL were transferred to a serum-gel tube for analysis of serum folate and ferritin ultrasensitive CRP; and the remaining blood (4 mL) was transferred to a zinc-free tube for determination of serum zinc.

Analysis of folic acid and ferritin were performed by the chemiluminescence method. Serum zinc by flame atomic absorption spectrophotometry (FAAS) using a PerkinElmer spectrometer model 3110 (PerkinElmer, Norwalk, CT, USA). The following measurements’ conditions were used: air-acetylene flame, 285.2 nm wavelength, 0.7 nm slit, and single-element HCL lamps. Gas flow and burner position were optimized before measurements to achieve high sensitivity. The samples were diluted appropriately to fit into the linear range of calibration curves. The lowest concentration traceability for zinc was 0.5 µg/L. Despite dilution, no sample pre-treatment procedures were applied prior to quantitative elements determination. Depending on the total sample volume, triplicate determinations were performed. The accuracy of FAAS technique was tested by means of recovery analysis, which for Zn was in the range of 94%–99%. All reagents used were of analytical grade. The test tubes for Zn were thoroughly acid washed (0.1% nitric acid) and rinsed with double distilled deionized water. Erythrocyte thiamin (thiamine diphosphate) was analyzed by High Performance Liquid Chromatography (HPLC) according to Mancinelli *et al*. [16].The acute phase proteins C-reactive protein (CRP) was measured by immunoturbidimetry. The mean corpuscular volume (MCV), mean corpuscular hemoglobin (MCH) and mean corpuscular hemoglobin concentration (MCHC) were calculated as the ratio betweenhemoglobinand red blood cells.

Laboratory cut-offs used for each parameter were as follows: low erythrocyte <3.9 millions/mm^3^, low hemoglobin <11.4 g/dL, low hematocrit <34%, low MCV <75 fL, low MCH <24 pg, low MCHC <31%. Low iron stores was defined as ferritin<15 µg/L; folic acid deficiency was defined as folic acid <3.0 ng/mL; zinc deficiency was defined as serum zinc <30.0 µg/dL and thiamine deficiency was defined as erythrocyte thiamine <28 µg/L. CRP >5 µg/L was used as an indicator for an acute phase response by infection or inflammation. All cut-offs were provided by the Laboratory of Clinical Analyses, Division of Health of the Federal University of Viçosa (UFV), which was also responsible for the quality control of the methods used.

### 2.5. Dietary Assessment

Consumption by the children was evaluated by the method of direct weighing of foods included in the meals served in the preschools, complemented with a 24-h recall of food intake at home, conducted the day after direct weighing. To control the intrapersonal variability, a second weighing was conducted with 25% of the children, selected randomly. Data collection was performed by trained interviewers.

The analyses and calculations of the diets were performed using the Avanutri software, version 2.0. Energy, calcium, iron, zinc, sodium, vitamins A, B1, B2, B12, C and folate were estimated. The adequacy of nutrients was evaluated by the Dietary Reference Intakes (DRI), characterizing the intake as above or below the Estimated Average Requirement (EAR), Adequate Intake (AI) and Tolerable Upper Intake Level (UL) [14,16,17,18] for children aged 1 to 3 and 4 to 8 years old (Table 1). Energy intake was calculated according to the Estimated Energy Requirements (EER). To calculate the EER, the physical activity factors used were of mild activity (1.13 for boys and 1.16 for girls). In the case of children who practiced some physical activity in addition to the usual activities, the active physical activity factor was considered (1.26 for boys and 1.31 for girls).

For practical application purposes in the analysis of school meals, the average intake was compared with the RDA values, which constitutes a parameter for the individual intake goal.

### 2.6. Determination of the Healthy Eating Index (HEI)

The HEI has been adapted to the Brazilian population [19] by adopting the Food Guide for the Brazilian Population [20] as a reference in addition to the proposal of Previdelli *et al*. [7] (Table 1). The components of the HEI assess adequacy (food groups and oils) and moderation (nutrient intake), with adjustment for energy density (per 1000 kcal).

Because the recommendation of the Brazilian Food Guide [20] is for a diet of 2000 kcal, the recommended number of servings was divided by 2 to perform this adjustment. For “SoFAAS components” (calories from solid fats, sugar and alcohol), the cutoff points suggested by Previdelli *et al*. [7] were used (Table 1).

The total score of the HEI ranges from 0 to 100, where the closer to 100 the better the quality of the diet. Each component was scored so that it assigned maximum values(5, 10 or 20 points) for meeting the recommendations and the minimum value (0 point) for no consumption and inadequacy of nutrients/calories, with intermediate scores (8 points) for the components “sodium” and “saturated fat”. When consumption was between the minimum and the recommended values, calculation of the matching score was performed as suggested by Guenther *et al*. [19] (Table 2).

### 2.7. Statistical Analysis

The database was developed with the aid of the Excel software (Microsoft Excel 2000). Statistical tests were performed using the SigmaPlot program—Scientific Graphing and Data Analysis Software, version 11.0 (Systat Software, Inc., London, UK). The Kolmogorov–Smirnov test was used to assess the normality of the data. To analyze possible differences between the median or average of the variables in the two preschools, the Mann-Whitney test or *t*-test was used, with a significance level of 5% (*p* < 0.05). To analyze the effect of intervention with UR^®^, and biochemical data collected at the beginning and end of the intervention the Wilcoxon test was used for comparison when there was no normal distribution of the data, and by the paired *t*-test when the distribution was normal, using a significance level of 5% (*p* < 0.05).

### 2.8. Ethical Aspects

The study was approved by the Ethics Committee on Human Research of the Federal University of Viçosa (UFV) (Of. Ref. No. 061/2011). Caretakers signed the consent form, authorizing the child participation in the study.

## 3. Results

### 3.1. Sample Characterization

Of the 99 preschool children in the study, 53.5% (*n* = 53) were female, 47.5% (*n* = 47) were between two and three years old and 52.5% (*n* = 52) were four to six years old.

### 3.2. Laboratory Tests

At baseline, children in both groupswerehomogeneousin relation toconcentrations oferythrocytes, hemoglobin,hematocrit, mean corpuscularvolume (MCV), mean corpuscularhemoglobin (MCH), mean corpuscular hemoglobin concentration (MCHC), folic acid,thiamine, serum zincand C-reactive protein (CRP)(*p* > 0.05).

At the end of the study, the values of MCH (*p* < 0.001), MCHC (*p* < 0.001), folate (*p* < 0.003) and serum zinc (*p* < 0.001) increased when compared to the beginning of the study in the test group (Table 3). In both groups erythrocyte thiamine concentration increased at the end of the study (*p* < 0.001). Serum ferritin concentration was higher at the end of the study in the control group (*p* < 0.001). At the beginning of the study, children in the test group showed higher concentrations of erythrocyte thiamin (*p* = 0.012) and ferritin (*p* < 0.001) (Table 3).

### 3.3. Consumption and Prevalence of Inadequate Nutrient

The insertion of UR^®^ in the diet improved the intake of micronutrients, since the intake of thiamine, folic acid and iron, nutrients present in the UR^®^ was higher in both age groups of the test preschool (Table 4). The two- and three-year-old children from the test preschool also showed higher intake of riboflavin and calcium, while four- to six-year-old children consumed more vitamin A and calcium than children of the control preschool. Regardless of age, there was a greater intake of vitamin C and sodium in the control preschool.

**Figure 2:**
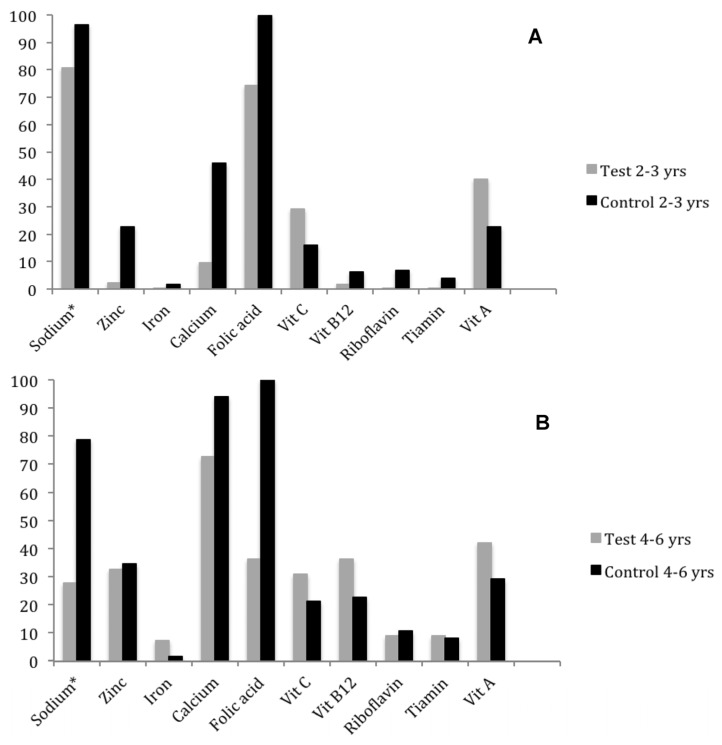
Percentage of inadequate ingestion of micronutrients in 2–3-year-old (**A**) and 4–6-year-old (**B**) preschoolers from two philanthropic preschools.

UR^®^ seemed to have improved zinc intake in children from the test preschool, even without significant changes in zinc consumption between preschools (Table 4, Figure 2). Regardless of age, the highest percentages of inadequate sodium intake were found in the control preschool. Among 4–6-year-old preschool children, folate intake inadequacy reached 100% in the control preschool and 36.32% in the test preschool (Figure 2).

### 3.4. Comparison of Iron, Zinc, Vitamin and Folic Acid Intake with the Individual Consumption Target

Insertion of UR^®^ contributed to reduce the percentage of two- to three-year-old children with folic acid consumption below the RDA (150 mg/day) in the test preschool (33.5%) more than the control preschool (65.5%). On average, iron, zinc and thiamine consumption of preschool children in this age group was found above the RDA [14,17] in both preschools. For 4–6-year-old children from the test preschool, the average consumption of iron, zinc, thiamine and folic acid was above the RDA [14,17]. Children from the control preschool, in turn, showed average consumptions of iron and folic acid 12.2% and 72.8% below the RDA, respectively. The average consumption of zinc and thiamine by children from the control preschool for this age group was above the RDA (Table 4).

### 3.5. Diet Quality Assessment of Preschool Children

The average total HEI scores were 57.2 and 57.3 in the test and control preschools, respectively, showing no difference between them (*p* = 0.936).

Scores of the components “dairy products” and “sodium” were greater for children of the test preschool, while for the control preschool higher scores were observed for “total fruit”, “dark green and orange vegetables” and “oils”. The components “whole grain”, “whole fruit” and “total fruit” were highlighted by the high percentage of zero scores, while “meat, eggs and legumes” and “dairy products” obtained a high percentage of the maximum score in both preschools (Table 5).

The scores for “total fruit” and “whole fruit” in both preschools were low, below half of the recommendation. Despite the higher score of “total fruit” from the control preschool (*p* = 0.011), 45.1% of preschool children did not consume foods of this group (Table 5). Regarding “total grain”, the mean was close to the maximum, and did not differ between preschools (*p* = 0.055). This component was composed entirely of refined grains, because 100% of preschool children presented a zero score for “whole grains”. For the group “oil”, the maximum score was achieved by 70.6% of children from the control preschool and 33% of children from the test preschool (Table 5). Regarding “saturated fat”, 16.7% of children from the test preschool obtained a score of zero, *i.e.*, consumption was higher than recommended, while in the control preschool 11.8% of children presented a maximum score for this component. “SoFAAS” received a maximum score from 4.2% children in the test preschool and 2% in the control preschool (Table 5).

## 4. Discussion

The UR^®^ improved the nutritional status of preschool children in relation to the concentrations of zinc, thiamin, folic acid, MCH and MCHC. The test group showed higher intake of vitamin B_1_, folate, calcium and iron when compared to the control group, which presented higher vitamin C intake in both age groups. Greater intake of vitamin B1, iron and folate in the test group may be attributed to the fortified rice (UR^®^) consumption, since this product is enriched with these micronutrients. Although the UR^®^ is also fortified with zinc, there was no difference regarding mean intake of this mineral between the test and control groups, due to greater consumption of foods considered sources of zinc by children of the control group. It is important to note thatonlythe intakeoffortifiedricewas monitored, and the school menusandhomemealswere not controlledduringthe intervention period.

Erythrocytes, hemoglobin and hematocrit were similar in both groups between the beginning and end of the intervention period. This can be explained by the adequate nutritional iron status of the children, *i.e.*, they did not present anemia at the beginning of the experiment. When iron levels absorbed by the diet are adequate, the intestinal mucosa regulates its absorption to maintain a constant amount of iron in the body, which would explain the similar values of the variables described previously [21]. However, values of MCH and MCHC increased after consumption of fortified rice by children of the test group, indicating a possible reduction of hypochromic red blood cells, and therefore any effect of fortified rice on the nutritional status of iron among these children.

At the beginning of the study, thiamine concentrations of the control group were lower than those of the test group. Thus, even without the use of fortified rice in school meals of the control preschool, improved nutritional status of this nutrient was observed possibly due to its increased uptake at the intestinal level.

These findings corroborate with the results observed in the dietary assessment, since children of the test group showed greater intakes of vitamin B_1_, folate and iron. Although children of the test group presented improved nutritional status of zinc at the end of the intervention period, the intake of this mineral did not appear different between the evaluated preschools.

The serum ferritin concentration was higher at the end of the study in children of the control group. Analysis of food consumption revealed factors that may explain the higher iron stocks in children of the control preschool, such as the intake of calcium, and vitamins A and C. Calcium intake was higher in the test preschool children. Although calcium has a negative influence on iron bioavailability, the mechanism by which dietary calcium reduces iron absorption is still poorly understood. There is some competitive inhibition between calcium and iron in the final transport of intestinal mucosa to the plasma, which occurs for heme and non-heme iron [22].

Vitamin A intake was highest among children between four and six years old in the test group, which may be another possible explanation for the higher iron stocks in these children at the end of the study. Vitamin A has an effect on the iron metabolism; however, this mechanism is not completely understood. It is possible that this vitamin interferes with the mobilization of available iron stocks and the use of iron to form hemoglobin [23], *i.e.*, the concentrations of ferritin may be reduced in children with greater consumption of vitamin A.

Consumption of vitamin C, which was higher in children of the control group, is also a factor that may explain the higher ferritin concentrations in these children. When this vitamin is present in adequate amounts, its rapid effect on the iron absorption percentage is noted [24].

Serum ferritin concentrations were higher before intervention among children in the test group, which may also explain the more evident increase in relation to this variable in children of the control group, whose ferritin levels were lower.

The results showed that the UR^®^ may be a good strategy of food fortification, reducing the inadequate intake of essential micronutrients. For example, higher intake of thiamine, iron and folic acid by children from the test preschool, in both age groups, may be assigned to the consumption of fortified rice (UR^®^), since this product is enriched with these micronutrients.

Although UR^®^ is also fortified with zinc, there was no difference in the average intake of this mineral among preschoolers, since the inadequacy percentage (Figure 2) in the test preschool (2.28%) was much lower than in the control preschool (22.66%). This difference can be explained by the higher standard deviation of the control preschool (SD = 5.93 mg) compared to the test preschool, since its calculation is based on the formula: (EAR − Mean)/Standard Deviation. The high standard deviation value of the control preschool can be explained by food consumption at home, which is more diverse than that offered in the preschool.

Even with the reduction in percentage of inadequate intake, folic acid intake by many preschoolers is still insufficient, making it necessary to increase the intake of foods considered sources of this vitamin, such as viscera, beans, dark green leafy vegetables and fruits like avocado, orange and apple [25]. It is also suggested to increase the proportion of UR^®^ mixed with polished rice (e.g., 1:50), thus reducing the inadequacy of folic acid without increasing the concentration of other micronutrients to above the upper level (UL).

Although preschoolers are not considered a risk group for deficiency of folic acid and thiamine, the importance of these micronutrients in the iron metabolism should be emphasized, and consequently in reducing the prevalence of iron deficiency anemia. Folic acid is an essential nutrient for maintenance and normal erythropoietic function and is essential in several cell metabolic reactions, vital to functioning of the body and normal growth [26]. Thiamine participates as an enzyme cofactor in the energy metabolism, especially that of carbohydrates and branched chain amino acids, greatly contributing to infant growth and development [27].

Zinc deficiency, in turn, is considered a nutritional problem worldwide, which according to some studies may be as frequent as that of iron. Deficiencies can coexist, due to the similarity of food sources and the factors that prevent the absorption of these minerals [28]. In addition to aiding in zinc deficiency prevention, the presence of this mineral in the fortified rice can also be justified by its important role in iron metabolism, acting as a regulator of divalent metal transporters at the intestinal level, thus increasing its absorption [28].

The interaction between some micronutrients in the diet may benefit or hinder the absorption of vitamins and minerals present in UR^®^. Among the positive interactions, one can mention the increased iron bioavailability generated by concomitant intake of calcium diets rich in vitamin C and vitamin A. Vitamin A increases the gene expression of proteins involved in iron metabolism, thus improving its uptake [29]. Vitamin C, in turn, appears to participate in regulation of the iron metabolism, increasing its absorption and modulating gene expression of ferritin and transferrin receptors [30]. On the other hand, the simultaneous use of high doses of calcium and iron affects iron absorption, since they compete for the same absorption sites [31].

Overall, there was greater consumption and lower prevalence of iron, thiamine and folic acid inadequacy of children in the test preschool that consumed UR^®^, regardless of the age group studied. Because the UR^®^ costs only 5% more than traditional rice [32], its inclusion in school meals may be an interesting strategy when taking into account the benefits it provides, such as increasing the daily intake of essential micronutrients at a reduced cost.

This study also sought to evaluate the diet quality of preschool children so as to evaluate the possible measures to be taken to correct inadequacies. It was found that the diet of preschool children had low total scores for the HEI, *i.e.*, eating was considered inappropriate and somewhat varied. A study by Hiza *et al*. (2013) [33] with two- to five-year-old American children showed similar results, with a mean score of 56. Mañios *et al*. (2009) [30], in a study in Greece with 2287 preschoolers, found an average score of 58.2 for two- to three-year-old children and 59.9 for four- to five-year-old children. These data demonstrate the need for changes in global infant eating habits.

The reduced intake of fruits and vegetables, and a lack of consumption of whole grains because of their higher prices or lower sensory acceptance may have contributed to the low consumption of fiber and micronutrients, again demonstrating that the inclusion of fortified rice may be considered an interesting strategy to control the deficiencies of vitamins and minerals.

It was observed that the component “meat, eggs and legumes” was well punctuated in both preschools, because even when meat consumption was low, bean consumption was increased. Legumes are not only inserted in the meat group because, when in surpluses, they are included in “total vegetables” and “dark green and orange vegetables”, potentially overstating the actual consumption of these foods, being considered a limitation of studies with the HEI. It is highlighted that the consumption of legumes may have favored the scoring of three of the twelve components of the HEI, which indicates the need for adaptation of this component in the HEI-2005 to the Brazilian population, since daily bean consumption is part of this culture [20].

For the group “oils”, which includes monounsaturated fats, oil from nuts and fish fats [19], the maximum score was achieved by approximately 70% of children from the control preschool and 33% of children from the test preschool. Because the scoring of the HEI does not include inverse values for those that surpass the recommendation, achievement of the maximum score in this component will not always be beneficial, since these are high-calorie foods that require moderate consumption [34].

It was observed that the sodium inadequacy percentage was very high in both preschools, mainly in the control preschool. The test preschool showed 2.1% of maximum scores and the control preschool is highlighted by the high percentage of zero scores (45.1%). Chronic consumption of high quantities of sodium can lead to complications such as increased blood pressure and mortality from cardiovascular diseases, even in children [35,36]. The component “SoFAAS” (solid fats and added sugar) showed low scores in the two preschools, indicating frequent use. The average score of the component “saturated fat” was not very low and did not differ between the preschools, demonstrating that simple sugars contributed significantly to the low score of “SoFAAS”. According to the Food Guide for the Brazilian Population [37], simple sugars should be used in small amounts since their excess coupled with a sedentary lifestyle has been associated with obesity, diabetes and cancer [38,39].

This was the first study to evaluate the impact of fortified rice on the diet quality and biochemical measurements in non-anemic children, which makes the comparison with other studies a difficult task. More crossover studies are needed to assess the impact of using UR^®^ on diet quality and biochemical variables of preschool children, reducing the bias especially in relation to differences in baseline micronutrient values.

## 5. Conclusions

The UR^®^ improved the nutritional status of preschool children in relation to the concentrations of zinc, thiamin, folic acid, MCH and MCHC. The inclusion of UR^®^ promoted improved micronutrient intake, especially thiamine, iron and folic acid. This product can be considered a good strategy for food fortification since it is inexpensive and provides approximately one-third of the recommended daily amount of iron, zinc, folic acid and thiamine in each typical rice portion. Their inclusion in official school lunch programs constitutes an important tool in the control of micronutrient deficiencies at the population level. However, although the UR^®^ increased the intake of micronutrients, quality and distribution of food within the food groups should be enhanced, also emphasizing the importance of food and nutrition education practices among preschoolers and those responsible.

## Figures and Tables

**Table 1:** Adaptation of the methodology for assessment the Healthy Eating Index (HEI) for the Brazilian population.

Component	Characteristic	Proposal HEI, 2005 *	Recommendation of the Brazilian Food Guide ** (in 2000 Kcal)	Recommendation of the Brazilian Food Guide *** (in 1000 kcal)
**1. Total fruit**	Includes whole fruits and juice	Recommendation of fruits’ servings/1000 kcal	3 servings	1.5 serving
**2. Whole fruits**	Excludes juice	Half of fruits’ recommendation/1000 kcal	1.5 serving	0.75 serving
**3. Total vegetable**	Includes all vegetables. Legumes are part of this group only if the meat group’s recommendation is reached.	Recommendation of vegetables’ servings/1000 kcal	3 servings	1.5 serving
**4. Dark green and orange vegetables and legumes**	Includes only dark green and orange vegetables. Legumes are part of this group only if the meat group’s recommendation is reached.	Daily sum of My Pyramid’s recommendations for the subgroups of dark green and orange vegetables/1000 kcal. Approximately 1/3 of the recommended servings for total vegetables/1000 kcal	1.5 serving	0.75 serving
**5. Total grains**	Includes all foods from the grain group.	Recommendation of grain servings/1000 kcal	6 servings	3 servings
**6. Whole grains**	Includes only whole grains and derivatives.	Half of the grain recommendation/1000 kcal	3 servings	1.5 serving
**7. Dairy products**	Includes milk and dairy products and soy based products.	Recommendation of milk and dairy product servings/1000 kcal	4 servings	2 servings
**8. Meat, eggs and legumes**	Includes meat, eggs and legumes.	Recommendation of meat servings/1000 kcal	2 servings	1 serving
**9. Oil**	Includes vegetable oil and unhydrogenated vegetable oil, fish oil, nuts and seeds.	Recommendation of oil servings/1000 kcal	1 serving	0.5 serving
**10. Saturated fat**	Corresponds to the percentage of total calories from the saturated fat.	Minimum limit (7%), intermediate (10%) and maximum (15%), according to the national recommendations.	Minimum limit (7%), intermediate (10%) and maximum (15%)	-
**11. Sodium**	Corresponds to the ingestion of the mineral sodium in milligrams.	Estimative considering the sodium recommendation and the medium ingestion of energy.	Minimum limit (700 mg/1000 kcal), intermediate (1100 mg/1000 kcal) and maximum/1000 kcal)	-
**12. Calories from solid fats, sugar and alcohol (SoFAAS)**	Corresponds to the calories from solid fat (saturated and trans in milk and dairy products, meat, butter, lard, chocolate); sugar (used in recipes and added) and alcoholic beverages.	Minimum limit corresponds to the percentile 86 (50%) and maximum limits for the 10 thpercentile (20%)	-	Minimum limits corresponds to the percentile 86 (10%) and maximum limits to the percentile 16 (35%) of the population

Source: * GUENTHER *et al*. (2007) [19]; ** BRASIL (2006) [20]; *** PREVIDELLI *et al*. (2010) [7]. Highlighted in gray are the HEI components, the American proposal and the adaptation used in this study. The recommendations are daily.

**Table 2:** Adaptation of the distribution of the scores of the components of the Healthy Eating Index.

Components	Minimum Score (0)	Intermediate Score (8)			Maximum Score (5 *, 10 ** or 20 ***)
Total fruit ^1^	0	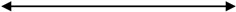			1.5 serving/1000 kcal *
Whole fruit ^1^	0	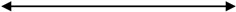			0.75 serving/1000 kcal *
Total vegetable ^1^	0	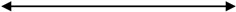			1.5 serving/1000 kcal *
Dark green and orange vegetables and legumes ^1^	0	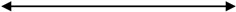			0.5 serving/1000 kcal *
Total grains ^1^	0	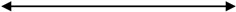			3 servings/1000 kcal *
Whole grains ^1^	0	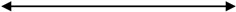			1.5 servings/1000 kcal *
Dairy products	0	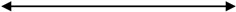			1.5 serving/1000 kcal **
Meat, eggs and legumes	0	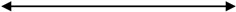			1 serving/1000 kcal **
Oil ^1^	0	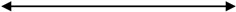			0.5 serving/1000 kcal **
Saturated fat ^1^	≥15% of TEV	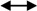	10% do VET	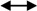	7% of TEV **
Sodium ^1^	≥2 g/1000 kcal	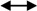	1.1 g/1000 kcal	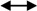	≤0.7 g/1000 kcal **
SoFAAS^2^	≥35% of TEV	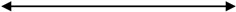			≤10% of TEV ***

^1^ Adapted from Guenther *et al*. (2007) [19]; ^2^ Adapted from Previdelli *et al*. (2010) [7], TEV = Total Energy Value. *: Maximum score = 5; **: Maximum score = 10; ***: Maximum score = 20.

**Table 3:** Biochemical variables of preschool children in the test and control groups, before and after the intervention.

Variables	Test Preschool				Control Preschool				
	Before Intervention	After Intervention	Difference	*p* ^a^	Before Intervention	After Intervention	Difference	*p* ^a^	*p*Baseline ^b^
Erythrocytes (millions/mm^3^)	4.90 ± 0.32 (4.92)	4.85 ± 0.36 (4.87)	−0.04 ± 0.27 (−0.04)	0.305	4.86 ± 0.42 (4.95)	4.90 ± 0.34 (4.92)	0.04 ± 0.29 (0.00)	0.367	0.670
Hemoglobin (g/dL)	12.34 ± 0.80 (12.10)	12.54 ± 0.88 (12.30)	0.19 ± 0.64 (0.00)	0.111	12.40 ± 0.87 (12.30)	12.57 ± 0.95 (12.50)	0.18 ± 0.64 (0.10)	0.079	0.781
Hematocrit (%)	39.27 ± 2.27 (39.05)	39.14 ± 2.82 (38.60)	−0.13 ± 2.16 (−0.20)	0.696	38.87 ± 2.68 (38.40)	39.17 ± 2.62 (38.75)	0.29 ± 2.21 (0.25)	0.334	0.464
MCV (fL)	80.33 ± 4.29 (80.94)	80.66 ± 3.95 (81.54)	0.32 ±1.24 (0.27)	0.097	80.20 ± 4.81 (80.56)	80.05 ± 4.71 (79.54)	−0.16 ± 2.16 (0.30)	0.628	0.769
MCH (pg)	25.22 ± 1.53 (25.59)	25.74 ± 1.53 (26.08)	0.52 ±0.63 (0.60)	**<0.001**	25.59 ± 1.95 (25.90)	25.61 ± 1.86 (25.53)	0.02 ± 1.02 (0.16)	0.223	0.314
MCHC (%)	31. 37 ± 0.56 (31.40)	31.87 ± 0.66 (31.85)	0.50 ± 0.73 (0.45)	**<0.001**	31.89 ± 0.78 (31.99)	31.97 ± 0.68 (32.20)	0.08 ± 0.77 (0.22)	0.079	0.866
Folic acid (ng/mL)	17.62 ± 3.08 (19.50)	19.72 ± 5.08 (21.00)	2.10 ± 5.99 (3.20)	**0.003**	17.38 ± 5.28 (17.35)	17.58 ± 4.23 (17.35)	0.20 ± 5.11 (0.00)	0.796	0.962
Thiamine (µg/L)	72.12 ± 25.85 (66.67)	194.76 ± 93.33 (145.84)	122.64 ± 98.62 (85.11)	**<0.001**	58.29 ± 16.51 (53.69)	101.19 ± 61.23 (95.19)	42.89 ± 33.50 (37.19)	**<0.001**	**0.012**
Ferritin (ng/mL)	32.80 ± 15.89 (30.65)	34.17 ± 16.10 (28.45)	1.82 ± 14.50 (2.75)	0.082	12.38 ± 0.84 (12.30)	36.45 ± 15.34 (34.50)	24.07 ± 15.53 (21.70)	**<0.001**	**<0.001**
Serum zinc (µg/dL)	80.97 ± 11.88 (81.40)	110.21 ± 22.76 (108.80)	29.25 ± 23.31 (26.30)	**<0.001**	95.86 ± 21.90 (95.40)	92.13 ± 15.92 (92.10)	−3.73 ± 27.76 (−7.20)	0.378	0.070
CRP (mg/dL)	0.26 ± 0.59 (0.01)	0.12 ± 0.32 (0.02)	−0.15 ± 0.44 (0.00)	0.128	0.36 ± 1.06 (0.03)	0.34 ± 0.57 (0.10)	−0.02 ± 1.15 (0.01)	0.226	0.516

MCV: mean corpuscular volume; MCH: mean corpuscular hemoglobin; MCHC: mean corpuscular hemoglobin concentration; CRP: C-reactive protein. Results obtained by biochemical analysis, expressed in mean ± standard deviation, median (minimum and maximum) of variables of two- to six-year-old children after four months of study. *n* = 99 children (49 in test preschool and 50 in the control preschool). Bold values: *p* < 0.05. ^a^ *p* values for comparison of variable medians in the same preschool, before and after intervention period (Wilcoxon test (non-parametric distribution)); ^b^ *p* values for comparison of variable medians between the two preschools in the beginning of the study (Mann-Whitney test (non-parametric distribution)).

**Table 4:** Comparison of the ingestion, at home and at the preschool, of micronutrients between preschoolers from two philanthropic preschools.

Variables	2 to 3 Years Old			4 to 6 Years Old		
	Test Preschool	Control Preschool	*p*	Test Preschool	Control Preschool	*p*
	Mean ± SD	Mean ± SD		Mean ± SD	Mean ± SD	
	Med (Min-Max)	Med (Min-Max)		Med (Min-Max)	Med (Min-Max)	
Vitamin A	276.82 ± 621.66	304.93 ± 125.47	0.090	435.41 ± 893.06	392.43 ± 207.05	**0.040**
(RE) *	206.12 (40.21–1300.38)	271.72 (94.55–561.74)		248.04 (38.74–4994.90)	367.61 (117.70–947.14)	
Thiamine	1.28 ± 0.33	0.96 ± 0.32	**0.003**	2.70 ± 1.60	1.19 ± 0.49	**<0.001**
(mg) **	1.28 (0.69–2.14)	0.91 (0.31–1.75)		2.18 (0.98–7.53)	1.04 (0.75–2.71)	
Riboflavin	1.49 ± 0.40	1.09 ± 0.46	**0.004**	1.42 ± 0.69	1.13 ± 0.51	0.052
(mg) **	1.48 (0.53–2.32)	1.04 (0.28–2.00)		1.36 (0.44–3.51)	1.02 (0.44–2.50)	
Vitamin B_12_	2.75 ± 0.97	2.39 ± 1.10	0.244	4.33 ± 9.75	2.18 ± 1.61	0.063
(µg) **	2.87 (0.63–5.20)	2.21 (0.89–5.20)		2.25 (1.14–54.67)	1.94 (0.28–8.28)	
Vitamin C	48.64 ± 66.36	64.79 ± 50.63	**0.006**	36.34 ± 28.23	99.66 ± 94.73	**<0.001**
(mg) *	24.09 (11.08–322.76)	50.81 (21.54–221.74)		29.66 (6.66–128.62)	74.79 (16.86–456.33)	
Folic acid	99.68 ± 31.05	51.78 ± 22.99	**<0.001**	218.19 ± 170.54	54.34 ± 24.61	**<0.001**
(µg) *	92.46 (57.68–171.58)	43.87 (21.54–96.57)		160.79 (53.16–790.18)	44.91 (24.22–125.75)	
Calcium	827.62 ± 255.93	520.35 ± 250.55	**<0.001**	668.73 ± 220.91	495.70 ± 195.45	**0.004**
(mg) **	802.89 (494.62–1392.31)	518.78 (123.50–993.42)		601.91 (321.96–1306.57)	443.50 (190.08–908.68)	
Iron	8.94 ± 1.65	7.55 ± 2.18	**0.021**	10.34 ± 4.04	8.78 ± 2.19	**<0.001**
(mg) **	9.11 (5.52–11.81)	7.53 (3.61–12.20)		11.90 (5.92–13.52)	9.05 (4.60–12.50)	
Zinc	5.31 ± 1.39	6.98 ± 5.93	0.207	10.83 ± 14.53	9.22 ± 12.39	0.063
(mg) *	5.02 (3.23–8.30)	5.67 (3.27–33.70)		7.11 (3.13–83.60)	5.86 (3.97–65.30)	
Sodium	1934.95 ± 313.51	2551.54 ± 709.51	**0.001**	1750.76 ± 695.22	2948.56 ± 1083.02	**<0.001**
(mg) *	1951.70 (1320.60–2596.60)	2539.55 (1432.00–3934.30)		1581.35 (797.60–3986.50)	2636.05 (1647.20–5682.10)	

* Mann-Whitney Rank Sum test. ** *t*-test. Med = Median, Min = Minimum, Max = Maximum. Values in bold: *p* < 0.05.

**Table 5:** Comparison of the total scores and of the components of the Healthy Eating Index in children from two philanthropic preschools.

Variables	Test preschool			Control Preschool			*p*
	Mean ± SD	% Score Zero	% Maximum Score	Mean ± SD	% ScoreZero	% Maximum Score	
	Med (Min-Max)			Med (Min-Max)			
Total fruit *	0.9 ± 1.6 0.0 (0.0–5.0)	66.7	6.3	1.9 ± 2.0 1.8 (0–5.0)	45.1	13.7	**0.011**
Whole fruit *	0.8 ± 1.7 0.0 (0.0–5.0)	77.1	10.4	1.3 ± 1.9 0.0 (0.0–5.0)	62.7	9.8	0.155
Total vegetable *	2.8 ± 1.9 2.8 (0.1–5.0)	-	25.0	3.5 ± 1.5 3.9 (0.0- 5.0)	2.0	33.3	0.055
Dark green and orange vegetables and legumes	2.3 ± 1.8 2.0 (0.0–5.0)	12.5	22.9	3.5 ± 1.7 4.4 (0.0–5.0)	2.0	49.0	**<0.001**
Total grains *	4.0 ± 0.9 4.1 (2.2–5.0)	-	20.8	3.8 ± 0.9 3.9 (2.0–5.0)	-	14.6	0.235
Whole grains *	0,0 ± 0,0 0.0 (0.0–0.0)	100	-	0,0 ± 0,0 0.0 (0.0–0.0)	100	-	1.000
Dairy products *	9.1 ± 1.7 10.0 (3.7–10.0)	-	68.8	7.8 ± 2.8 9.3(1.9–10.0)	-	43.1	**0.006**
Meat, eggs and legumes *	9.1 ± 1.8 10.0 (1.3–10.0)	-	68.8	9.5 ± 1.1 10.0 (5.0–10.0)	-	68.6	0.795
Oil *	6.2 ± 3.4 6.2 (0.2–10.0)	-	33.3	9.3 ± 1.3 10.0 (5.0–10.0)	-	70.6	**<0.001**
Saturated fat *	6.4 ± 3.8 7.6 (0.0–10.0)	16.7	25.0	7.1 ± 3.8 9.1 (0.0–10.0)	11.8	35.3	0.255
Sodium *	5.3 ± 3.2 5.3 (0.0–10.0)	6.3	2.1	1.3 ± 1.8 0.3 (0.0–6.3)	45.1	-	**<0.001**
SoFAAS **	10.2 ± 5.1 10.3 (0.0–20.0)	4.2	4.2	8.4 ± 5.2 8.2 (0.0–20.0)	11.8	2.0	0.087
Total score *	57.2 ± 12.6 59.6 (34.0–77.2)	-	-	57.3 ± 10.7 59.5 (34.6–75.0)	-	-	0.936

* Mann–Whitney Rank Sum test (nonparametric distribution). ** *t*-test (parametric distribution).Med = Median, Min = Minimum, Max = Maximum. Values in bold: *p* < 0.05.

## Abbreviations

The following abbreviations are used in this manuscript:

AI	Adequate Intake
DRI	Dietary Recommended Intake
EAR	Estimated Average Requirement
EER	Estimated Energy Requirement
SoFAAS	Calories from Saturated Fat, Added Sugar and Alcohol
HEI	Healthy Eating Index
RDA	Recommended Dietary Allowances
SD	Standard Deviation
UL	Upper Level
UR^®^	Ultra Rice^®^
